# Do children with central venous line (CVL) dysfunction have increased risk of symptomatic thromboembolism compared to those without CVL-dysfunction, while on cancer therapy?

**DOI:** 10.1186/1471-2407-12-314

**Published:** 2012-07-26

**Authors:** Jacqueline Halton, Kim Nagel, Leonardo R Brandão, Mariana Silva, Paul Gibson, Anthony Chan, Kay Blyth, Kim Hicks, Nagina Parmar, Leslie Paddock, Stephanie Willing, Lehana Thabane, Uma Athale

**Affiliations:** 1 The Children’s Hospital of Eastern Ontario (CHEO), Ottawa, ON, Canada; 2 McMaster University/Hamilton Health Sciences, Hamilton, ON, Canada; 3 The Hospital for Sick Children, Toronto, ON, Canada; 4 Cancer Centre of Southeastern Ontario at KGH, Kingston, ON, Canada; 5 The Children’s Hospital at LHSC, London, ON, Canada; 6 Biostatistics Unit, St. Joseph’s Healthcare—Hamilton, Hamilton, ON, Canada

**Keywords:** Central venous line, Central venous line dysfunction, Infection, Thromboembolism, Cancer, Children

## Abstract

**Background:**

Thromboembolism (TE) and infection are two common complications of central venous line (CVL). Thrombotic CVL-dysfunction is a common, yet less studied, complication of CVL. Two retrospective studies have reported significant association of CVL-dysfunction and TE. Recent studies indicate association of CVL-related small clot with infection. Infection is the most common cause of non-cancer related mortality in children with cancer. We and others have shown reduced overall survival (OS) in children with cancer and CVL-dysfunction compared to those without CVL-dysfunction. Despite these observations, to date there are no prospective studies to evaluate the clinical significance of CVL-dysfunction and it’s impact on the development of TE, infection, or outcome of children with cancer.

**Study design:**

This is a prospective, analytical cohort study conducted at five tertiary care pediatric oncology centers in Ontario. Children (≤ 18 years of age) with non-central nervous system cancers and CVL will be eligible for the study. Primary outcome measure is symptomatic TE and secondary outcomes are infection, recurrence of cancer and death due to any cause. Data will be analyzed using regression analyses.

**Discussion:**

The overall objective is to delineate the relationship between CVL-dysfunction, infection and TE. The primary aim is to evaluate the role of CVL-dysfunction as a predictor of symptomatic TE in children with cancer. We hypothesize that children with CVL-dysfunction have activation of the coagulation system resulting in an increased risk of symptomatic TE. The secondary aims are to study the impact of CVL-dysfunction on the rate of infection and the survival [OS and event free survival (EFS)] of children with cancer. We postulate that patients with CVL-dysfunction have an occult CVL-related clot which acts as a microbial focus with resultant increased risk of infection. Further, CVL-dysfunction by itself or in combination with associated complications may cause therapy delays resulting in adverse outcome.

This study will help to identify children at high risk for TE and infection. Based on the study results, we will design randomized controlled trials of prophylactic anticoagulant therapy to reduce the incidence of TE and infection. This in turn will help to improve the outcome in children with cancer.

## Background

Cancer is the leading cause of disease-related death in North American children [1,2]. Intensive therapy has resulted in over 80% of cure-rates in children with cancer, but therapy-related toxicity can limit the dose intensification of chemotherapeutic agents and compromise the prospect for cure [3]. Thus, it is important to minimize or prevent therapy-related complications.

Thromboembolism (TE) is a common and potentially fatal complication in children with cancer with up to 16% prevalence of symptomatic and ~ 40% of asymptomatic TE [4-10]. A recent population-based study showed dramatic increase in the incidence of TE in hospitalized children in the USA [4]. Cancer is a major underlying disease in children with TE and accounts for ~ 40% of pediatric TE [4-7]. We estimate that the risk of TE in children with cancer is at least 600-times higher than that in the general pediatric population [8-10].

TE is associated with significant morbidity, mortality and financial burden. The majority of symptomatic TE in children with acute lymphoblastic leukemia (ALL) occurs in potentially fatal sites; ~ 50% in the central nervous system (CNS) in the form of arterial ischemic stroke or sinovenous thrombosis, 2% pulmonary embolism (PE) and 2% in the right atrium [8,9]. A recent population based study showed that children with cancer are at increased risk of recurrent TE. Development of TE in children with cancer also leads to prolonged hospitalization and increased mortality [4]. The average case fatality ratio from TE in children with ALL is reported to be 15% [8,9]. In addition, the development of TE interferes with the scheduled therapy and therapy interruptions are known to compromise cure rates [10,11]. The estimated direct medical care cost of adult TE in the USA is ~ $600 million/year [12]. Although similar estimates are unavailable for children, development of TE undoubtedly adds to the direct and indirect therapy cost.

Thus, TE is a growing and significant problem in children with cancer. TE can be treated with anticoagulant therapy. Hence it is important to have reliable methods for early diagnosis of TE. In addition, TE is preventable with anticoagulant prophylaxis, even in children with cancer [13,14]. To avoid mortality and morbidity related to TE it is important to identify children at high risk for development of TE. However, the risk factors predisposing children with cancer to TE are yet to be defined.

Central venous line (CVL) is the single most important risk factor for development of TE in children [15-29]. Long-term CVL has become an integral part of cancer-therapy in children. It has improved both the quality of care and quality of life in children with malignancy. CVLs are commonly used to deliver chemotherapy, blood products, parenteral nutrition and other intravenous therapies as well as facilitate repeated blood drawing essential for the care of these patients. However, CVLs are associated with significant complications leading to morbidity and mortality [16-29]. Two recent studies have identified a CVL-related complications rate of 40%-46% in children with cancer [18,19]. Infection and TE are the two most common and serious medical complications related to CVL and may necessitate CVL removal in ~ 20%-35% of tunneled CVLs [29].

CVL-related TE is associated with significant acute and chronic morbidity and mortality. Catheter occlusion (in 11-40% patients), loss of venous access, infection (22-40%), embolism to other vessels including PE (~13%), and subsequent development of post-thrombotic syndrome (15-35%) are commonly described morbidities in patients with CVL-associated TE [21,23,25,30]. As observed in a recent study, patients with CVL-related TE had shorter CVL life and increased number of CVLs compared to those without TE [19]. Further, CVL-related thrombi may promote TE at other sites in the body. In addition, CVL-related acute morbidities lead to loss of CVL and interruption of chemotherapy [19-31]. Using a retrospective cohort design we and others have shown an adverse impact of CVL dysfunction on survival of children with cancer [32,33].

### Significance of CVL dysfunction

CVL-dysfunction (also known as occlusion or malfunction) is a common, but relatively less studied complication of CVL. Various studies defined dysfunction as the inability to infuse fluids and/or withdraw blood and can be mechanical or thrombotic [17,18,22,32,34]. The thrombotic occlusion is thought to be resulting from small clot at the tip or surrounding the tip of the CVL. Dysfunction can be partial where infusion is possible but blood cannot be aspirated or complete with inability to either aspirate or infuse. In a prospective study of adults with cancer, Lee et al., have documented 11% prevalence of CVL-dysfunction; interestingly patients with CVL-dysfunction had a significantly higher risk of developing symptomatic CVL-related TE (OR 14.7, p<0.001) [34].

In comparison to adults, studies in children with cancer have reported much higher prevalence of CVL-dysfunction ranging from 21%-39% [17,18,32,33,35]. Although, the higher prevalence of CVL-dysfunction observed in these studies may be related to the smaller sample size and retrospective study-design, physiologically, children are likely to be more susceptible for CVL-dysfunction due to the smaller vascular dimensions and higher CVL to vessel diameter ratio [35,36]. In fact the observation of higher risk of CVL-related complications in younger children supports this notion [17-20]. Three retrospective studies (including ours) have evaluated the risk of TE in children with cancer and CVL-dysfunction; all three studies have shown significantly increased risk of TE in children with CVL-dysfunction [17,32,33,36].

We propose a prospective, multicenter, cohort study to evaluate the role of CVL-dysfunction as a predictor of TE and to study the impact of CVL-dysfunction on the frequency of infection and outcome of children with cancer. We will also evaluate the impact of CVL-related (type, insertion techniques and care guidelines) and patient-related (age, height, body mass index) factors on the incidence of CVL-dysfunction, TE and infection. We plan to conduct this study in five tertiary care pediatric oncology institutions. The participating institutions are members of the Pediatric Oncology Group of Ontario (POGO) and are responsible for the diagnosis and therapy of all children with cancer in the province of Ontario.

The primary research question is as follows:

Do children with CVL dysfunction have increased risk of symptomatic thromboembolism compared to those without CVL dysfunction, while on cancer therapy?

### Primary hypothesis

Children with CVL-dysfunction are at increased risk for diagnosis of symptomatic TE while on therapy for cancer compared to those without CVL-dysfunction.

### Secondary hypotheses

1. Children with cancer and CVL-dysfunction have an increased prevalence of infection compared to those without CVL-dysfunction. Presence of both CVL-dysfunction and infection increases the risk of TE in children with cancer.

2. Children with cancer and CVL-dysfunction have decreased OS and EFS compared to those without CVL-dysfunction.

### Aims

The overall objective of the study is to explore the relationship of CVL-dysfunction, TE and infection in children with cancer and to study the impact of CVL-dysfunction on overall outcome in children with cancer.

### Primary aim

To compare the risk of symptomatic TE in children with or without CVL-dysfunction while receiving therapy for cancer.

### Secondary aims

In children receiving therapy for cancer,

1. To compare the prevalence of infection at any site in children with or without CVL-dysfunction

2. To compare the five-year OS and EFS in children with or without CVL-dysfunction

3. To delineate the relationship of CVL-dysfunction, infection and TE

4. To evaluate the impact of different CVL-related (type, insertion techniques, care guidelines) and patient-related (age, height, body mass index) factors on the development of CVL-dysfunction, TE and infection

### Study rationale

#### Rationale for primary aim

CVL-related TE in children is difficult to diagnose. CVL-related TE could be symptomatic (with pain, edema, skin discoloration and dilated veins) or asymptomatic [37,38]. The presence of symptoms reflects the site of obstruction, size of obstruction and acuteness of obstruction. In children, especially in younger age group, the symptoms of TE are difficult to detect. Hence even a significant TE may go un-noticed. Journeycake et al., showed that only 5 of 21 children with CVL-related TE were symptomatic; 10 of 16 “asymptomatic” children had multiple CVL placements prior to the diagnoses of TE [17]. This observation highlights the importance of a reliable screening method for diagnosis of CVL-related TE.

Although compression ultrasonography (USG) is the diagnostic test of choice for suspected lower venous system deep venous thrombosis (DVT), it has poor sensitivity for the diagnosis of DVT in the upper venous system (central subclavian vein, brachiocephalic and superior vena cava) [37,38]. Within the thoracic cage, the non-compressibility of the vessel cannot be assessed due to the bony ribs [39]. Prospective studies have shown that bilateral venography is the most sensitive technique for diagnosis of upper venous system TE within the thoracic cage [37-40]. However, venography is an invasive, cumbersome and painful procedure, and it exposes children to excessive radiation. Hence venography cannot be used as a routine screening tool for evaluation of CVL-related TE in children with cancer. Magnetic resonance venography (MRV) is another non-invasive, safe and reliable method to diagnose DVT. Although safe, MRV is very costly, requires sedation or general anesthesia for younger patients making it unsuitable for screening purposes, and its sensitivity and specificity in children have not been well defined [40]. Further, CVL-related small clots such as fibrin sheath or ball valve thrombi (which are mostly responsible for CVL-dysfunction) may not be detected by USG or even linogram.

Thus, there is no reliable, safe, inexpensive and noninvasive screening tool for diagnosis of CVL-related TE in children. Hence one may have to consider surrogate markers as predictors of underlying TE. An ideal predictor will reliably identify patients with high a probability of underlying TE for whom evaluation by invasive tests will be justified. Considering available evidence, we propose that CVL-dysfunction may be a good predictor of underlying TE. If indeed the presence of CVL-dysfunction can predict TE then such patients could be evaluated further with the gold standard test of venography and treated with anticoagulation therapy. This in turn will reduce the risk of development of symptomatic TE and infection. However, the first step is to confirm the presence (and the strength) of association of CVL-dysfunction and TE in a large prospective study.

#### Rationale for secondary aim 1

Bacterial and fungal infections and sepsis are well-known complications in children undergoing intensive chemotherapy. Presence of CVL increases the risk of infections [17-19,41-43]. Two recent prospective studies conducted in children with cancer and hematological disorders reported infection rates of 0.87 and 1.7/1000 catheter-days [18,19]. In one study, 40% of patients had CVL-related infections and 44% of the episodes required CVL removal [43].

Although the association of CVL with infections has been well documented, only recently investigators have begun to explore the correlation of CVL-related infection with underlying local TE [42,43]. Prospective studies conducted in adult patients have shown that the local or systemic CVL-related infections are likely to increase the risk of TE in patients on therapy for cancer [42,43]. A retrospective study showed increased risk of thrombosis in children with cancer and documented infection [odds ratio (OR) 2.2, p = 0.016] [17]. So far there is no prospective study to evaluate the relationship of TE and infection in children with cancer.

#### Rationale for secondary aim 2

Deitcher et al., reported CVL-dysfunction in 28% children with brain tumors; children with CVL-dysfunction, but not with major TE, had a reduced overall survival [32]. Further, our results suggest that CVL-dysfunction is an independent risk factor for poor OS and EFS in children with cancer [33]. The poor outcome in children with cancer and CVL-dysfunction could be related to the interruption in therapy due to the loss of CVL or due to the associated complications like TE and infection. In addition, CVL-dysfunction may represent activation of coagulation system; a marker of poor prognosis in adults with cancer [32,33,36]. Thus, CVL-dysfunction probably reflects significant underlying pathology. Despite these observations there are so far no prospective studies to evaluate the clinical significance of CVL-dysfunction. Secondary aim 2 evaluates the relationship of CVL-dysfunction with OS and EFS. CVL-dysfunction may herald underlying undetected TE; the early diagnosis of which may prevent complications like overt TE, infection and therapy-delays. This in turn will improve the overall outcome in children with cancer.

#### Rationale for secondary aim 3

Although the three common complications of CVL namely CVL-dysfunction, infection and TE seem to be closely associated, the exact relationship is unclear. One retrospective study in children with cancer showed an increased risk of TE in association with CVL-dysfunction (OR 3.7, p = 0.001) and infection (OR 2.2, p = 0.016) whereas children having both dysfunction and infection had much higher risk of TE (OR 6.4, p<0.001) [17].

Presence of a local clot may lead to CVL-dysfunction as well as may act as a nidus of infection providing fertile ground for growth of the microorganisms. This may lead to either local, CVL-related infection or infection elsewhere in the body. CVL-dysfunction also needs frequent manipulations such as repeated attempts to infuse or withdraw blood; this may increase the chances of infection in immunocompromised patients. On the other hand, presence of infection can potentially initiate activation of coagulation and formation of TE in the vessel which is already damaged by CVL or by hyperosmolar infusions. The small clot developed in the process may progress to cause a significant TE either locally or distally elsewhere in the body. This clot may also lead to CVL-dysfunction.

**Figure 1:**
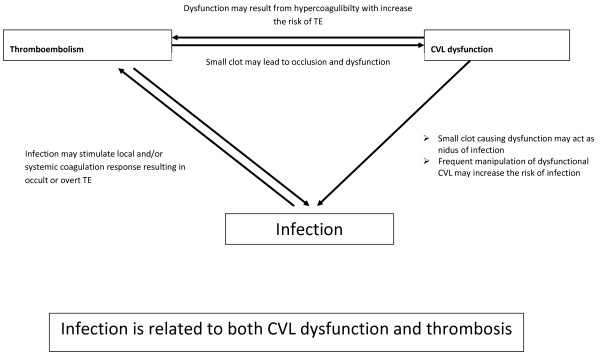
Possible association of infection, CVL dysfunction and thrombosis.

In summary, the cause and effect relationship of infection as well as the chronological relationship of infection, TE and CVL-dysfunction are still unclear. Figure 1 outlines our proposed hypothesis of the mechanism of interaction of these three CVL-related complications.

#### Rationale for secondary aim 4

Currently there are no uniform, standard guidelines for CVL insertion and care in children with cancer [44]. A survey conducted by United Kingdom Children’s Cancer Study Group showed that although 70%-80% of health care professionals thought that the CVL-dysfunction and TE were clinically important problems, there was remarkable variation in the diagnosis, management and prevention of CVL-dysfunction and TE [45]. There are no guidelines for ideal flush solution, its concentration or delivery schedule [23,44,45]. The CVL can be inserted either by an interventional radiologist with percutaneous technique or by the surgeons using cut-down technique. Although for an occasional patient the practice may vary, usually each institution has a consistent protocol for CVL-insertion. We conducted a survey of all five participating institutions evaluating the practice of CVL insertion and the CVL-care guideline. This survey showed that all the participating institutions use different guidelines for the care of the long term CVLs. In three of the five institutions usually surgeons insert the CVLs and in two institutions the interventional radiologists place the CVLs. By evaluating the impact of CVL- and patient-related factors on development of TE, infection and CVL-dysfunction as well as comparing the institutional practice of CVL insertion and care, we hope to develop evidence-based guidelines for CVL-care.

## Methods and design

**Figure 2:**
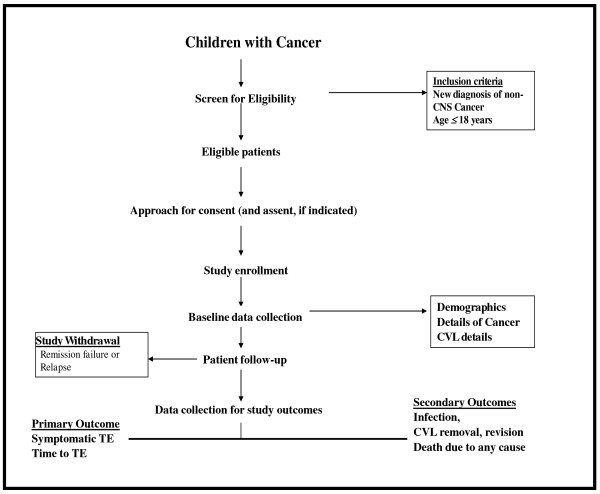
Flow diagram of the proposed study outlining the patient identification, study enrollment and follow up.

This is a prospective analytical cohort study conducted at five tertiary care pediatric oncology centers in Ontario. Figure 2 outlines the research design of the study.

### Justification for study design

Majority of children diagnosed with non-CNS cancer have CVL placed for therapy. Since both TE and infection are complications of cancer-therapy, we have chosen the best study design in observational methodology which is the prospective cohort study.

### Patient population

Children with non-CNS cancer diagnosed and treated at five participating institutions.

### Patient eligibility

#### Inclusion criteria

All children (≤ 18 years of age) newly diagnosed with non-CNS cancer will be eligible for the proposed study. Not all patients with CNS cancers receive CVL and the prevalence of TE in children with brain tumors is very low (< 2%) [32,36,46]. Hence we will include only children with non-CNS cancers.

#### Exclusion criteria

1. Patients with relapsed cancer since they would have had previous exposure to CVL

2. Age > 18 years at cancer diagnosis since majority are treated at the adult facilities

3. Absence of CVL

4. Diagnosis of CVL-dysfunction prior to study enrollment

5. Patients on anticoagulation therapy or prophylaxis

6. Unable or unwilling to provide written informed consent (and/or assent) for the proposed study

### Observations

#### Dependent variables

1. Development of symptomatic TE in any location while receiving therapy for cancer. Screening for asymptomatic TE will not be performed.

2. Clinical or microbiologically proven infection as defined below

3. Recurrence of cancer, second malignant neoplasm (SMN) or death due to any cause

#### Independent variables

1. CVL-dysfunction as defined below

2. Age of the patient at the time of diagnosis of cancer

3. Type of cancer

4. CVL insertion technique

#### Definitions

1. CVL-dysfunction: Persistent or recurrent difficulty in blood draw and/or infusion of at least one CVL lumen with or without the need for instillation of tissue plasminogen activator (tPA) documented on two or more occasions. For a multi-lumen CVL, difficulty in infusion or withdrawal of the same lumen for ≥ 2 occasions is required to fulfill the criteria of dysfunction.

2. Symptomatic thromboembolism: total or partial occlusion of one or more vessels objectively confirmed by at least one radiological diagnostic method prompted by typical clinical symptoms. CVL-related DVT: total or partial occlusion of vessel in which CVL is placed and/or right atrial TE.

3. Infection: Published guidelines will be used for diagnosis of CVL infection [47,48]. In addition a patient will be diagnosed with *infection* in any of the following situations:

For the purpose of this study patients with suspected or proven viral (e.g. influenza, chicken-pox) as well as mycoplasma infection will not be categorized as “infected”.

a. Septic shock requiring Pediatric Intensive Care Unit (PICU) admission with or without microbiological proof.

b. Positive blood culture collected from CVL and/or peripheral vein requiring intravenous (IV) antibacterial and/or antifungal therapy beyond 48 hours.

c. Invasive fungal (including candidial) infection defined as positive blood culture or positive culture from any other site (except oral or perianal candidiasis), radiological documented probable infection requiring systemic antifungal therapy and/or histopathological evidence of fungal infection (e.g. documentation of fungal hyphae on biopsy)

d. Clinically documented infection (e.g. CVL site infection, tunnel track infection, cellulitis, abscess formation at any site, typhlitis), with or without microbiological proof, requiring antibacterial or antifungal therapy beyond 48 hours. Minor infections like suspected otitis media, streptococcal throat infection will be excluded.

e. Persistent and/or recurrent fever of unknown origin requiring antibiotics therapy beyond 7 days.

4. Event: recurrence of cancer, SMN or death due to any cause.

#### Recruitment and consent process

Newly diagnosed cancer patients will be identified through pediatric oncology services at the participating institutions. Study staff will review patients’ records to determine eligibility for the proposed study. Eligible patients will be approached, prior to CVL placement or within two weeks of CVL placement, for informed consent. Reasons for non-participation will be recorded for all screened patients.

#### Study follow-up and study duration

This study will require no visits above and beyond those required for the clinical care of the patient. After initial hospitalization for evaluation and therapy for cancer, all patients are seen frequently (either weekly or monthly) at the outpatient clinics as part of their routine clinical care. Patients will be monitored for infection, disease progression or recurrence, or SMN. After completion of therapy patients are followed life-long according to the “After-Care-Guidelines” recommended by POGO.

Patient enrollment will occur until the required sample size is achieved. We anticipate that the required patient population will be enrolled during first two and half years of the study. Patients will be followed for the development of symptomatic TE until the completion of cancer therapy and final removal of CVL which occurs within 6–12 weeks post completion of therapy. Thereafter overall outcome data regarding continued remission, recurrence of cancer, SMN or death will be collected for each patient every six months for a total period of five years from the initial diagnosis.

#### Ethical considerations

##### Patient safety and inconvenience

This study will not pose any additional risks to the patients. Only pertinent data will be collected prospectively. No additional tests will be performed and there will not be any change in current institutional practice of CVL insertion, care or management of complications.

##### Ethics

This study is conducted in accordance with the Ethical Conduct for Research Involving Humans outlined in Tri-Council Policy Statement [49]. The study has been approved by the Research and Ethics Board (REB) of McMaster University and Hamilton Health Sciences as well as by the REB of each of the participating institution. Participation is fully voluntary after informed consent either from the patient or the parent/guardian as per the REB guidelines. In addition, assent will be obtained from patients 7–15 years of age (or younger if perceived competent to do so by physicians) as per institutional guidelines. Participants are informed about the nature of the study, their rights and obligations and are assured that refusal to participate does not affect patient care.

##### Confidentiality

Procedures are developed to protect the confidentiality of the data collected in accordance with the REB’s requirements and Canadian privacy legislation. All the data will be coded and stored securely to protect individual confidentiality.

##### Patient care and benefit

Patients with TE will be treated and counseled according to the recommended standard of care.

#### Data collection and analyses

##### Clinical data

Will be collected and recorded for all patients on standardized data collection form. In summary, data will include baseline patient characteristics, personal and family history of TE or prothrombotic disorder; diagnosis of cancer and cancer-therapy, details of CVL-dysfunction, details of infections and TE, if any, and overall outcome from cancer.

##### Details of CVL insertion

Details of CVL insertion (including the date, body side, type of CVL used, type of procedure, and any difficulty encountered) will be recorded. For patients requiring more than one CVL during the course of initial cancer therapy, details of each additional CVL will be recorded separately. The life span of the CVL will be measured in “catheter-days” calculated from the time of insertion and time of removal of CVL or the time of death or last follow-up if the CVL was still in place.

##### Diagnosis, evaluation and management of patients with symptomatic TE

To ensure uniformity of diagnosis and evaluation, uniform guidelines for the definition of symptomatic TE including clinical and radiological assessment at the time of diagnosis of TE as published elsewhere will be used. In addition, all patients diagnosed with symptomatic TE (either upper or lower limb or elsewhere in the body) are recommended to undergo bilateral upper venous system venography, contrast enhanced MRV or computerized tomography (CT) venogram to detect CVL-related TE. At the time of diagnosis of TE, laboratory evaluation will include CBC, coagulation profile (INR, APTT, D-dimer, fibrinogen) and measurement of prothrombotic defects as per institutional practice. Patients who develop a symptomatic TE will be managed according to the institutional guidelines. Clinical, laboratory and radiological details as well as management and outcome details of TE will be recorded on the data collection form.

##### Diagnosis, evaluation and management of patients with infection

Clinical and laboratory details of patients fulfilling the definition of “infection” for this protocol will be recorded. Patients with suspected or proven infection will be managed according to the institutional guidelines for the management of infection in an immunocompromised host.

##### Measurement of the exposure (i.e. CVL-dysfunction)

To avoid recall bias in recording CVL-dysfunction, CVL-access information will be collected and recorded prospectively. Currently on the the inpatient unit, every time the CVL is accessed, the nurse accessing the CVL completes the CVL checklist and records any problems in blood-draw, infusion or need for tPA. Similar checklist will be filled out for patients’ outpatient visits as well as emergency room visits, if any. These checklists will be collected and reviewed by the study staff at regular intervals In addition, during the regular clinic visit patients (or care givers) will be asked about the functioning of CVL and any problems encountered and their responses documented. We believe that combination of prospective documentation by nursing staff and patient recall will capture all the events of CVL-dysfunction. The patient will be categorized as “exposed” once the patient has met the criteria for CVL-dysfunction.

##### Data documentation and retrieval

All five participating institutions will use the CVL access and function check list. Our research staff will train the health care professionals for documentation and collection of primary and secondary outcome data. Data will be collected in real time by chart review and review of ancillary material (e.g. line function checklist).

##### Analysis plan

The analysis and reporting of the study will be performed in accordance with the STROBE Statement [50]. The primary outcome is a binary variable- symptomatic TE (presence/absence) as well as a continuous variable (time to event). Independent variables include: age, number of catheter days and time to CVL-dysfunction as continuous variables and, gender (male/female), cancer type, CVL-dysfunction (presence/absence), symptomatic TE (presence/absence), infection (presence/absence) as categorical variables. The process of participant recruitment and retention will be summarized using a flow diagram. Analysis results of participant recruitment and retention will be summarized using descriptive summary measures: mean (standard deviation) or median (minimum-maximum) for continuous variables; and number (percent) for categorical variables. We will use logistic regression analysis to determine the factors that can best discriminate between the two patient groups (i.e. those with and without TE). The list of potential factors is determined *a priori* and includes age of the patient at the time of diagnosis of cancer, cancer type; CVL-dysfunction (presence/absence), infection (presence/absence) and CVL type (internal/external). Univariate analyses will be performed to determine which variables to include in the multivariable models using the criterion of alpha = 0.20. Initially, we will use complete case analysis (i.e. do the analysis without taking missing data into account) and then repeat the analyses with missing data handled by multiple imputation technique [51-53].

For regression models, we will report the OR (for logistic regression), corresponding standard error, 95% confidence intervals and associated p-values. We will report P-values to 3 decimal places with p-values less than 0.001 reported as p<0.001. For all tests, we will use alpha =0.05 level of significance. Examination of residuals will provide an assessment of model assumptions for regression analyses. Goodnees-of-fit for the models will be performed using appropriate Hosmer-Lemeshov tests. For multivariable regression analysis, we anticipate multicolinearity [54]. We will assess colinearity using the variance inflation factor (VIF) which measures the extent to which the variance of the model coefficients will be inflated (because of the correlation of the variable with other predictor variables) if that variable is included in the model. Variables with VIF >10 will be considered colinear and will be excluded from the analysis [55].

The discriminant performance of the model will be evaluated using sensitivity, specificity and the area under the ROC – receiver operating characteristic curve. We will use bootstrapping approach to internally validate the model. We will compare the performance characteristics (i.e. sensitivity, specificity, area under the ROC curve, etc) between the original model and the bootstrap model. OS and EFS will be estimated using Cox-regression which will allow for adjustment of confounding variables. Hazard ratio (HR) will be calculated using log-rank method.

##### Sample size

The primary objectives of the study are to develop a model to discriminate between patients with and without symptomatic TE. To determine the optimal discriminant clinical model we will undertake a multivariable analysis. Simulation studies demonstrate that logistic models require 10 to 15 events per predictor to produce stable model estimates [54,55]. Based on our retrospective data and published reports we anticipate that at least 20% patients will be exposed (i.e. with CVL dysfunction) and 80% unexposed (i.e. without CVL dysfunction) [17,33,36]. Preliminary data from the retrospective study showed that the overall prevalence of TE was 11%; 23% of exposed and 9% of unexposed patients had TE. We will evaluate five predictors in our multivariable analysis. In this study, we have a fixed sample size of 450 patients (90 exposed with CVL dysfunction; and 360 without CVL dysfunction). The overall rate of symptomatic TE is assumed to be 11% (~50 events of TE) [36].

## Discussion

To our knowledge, this is the first prospective and comprehensive evaluation of CVL-dysfunction in pediatric oncology. This study will improve our knowledge regarding the mechanism of CVL-dysfunction and the relationship amongst CVL-dysfunction, infection and TE in children with cancer. Most importantly this study will evaluate the impact of CVL-dysfunction on the development of TE, infection and overall outcome from cancer in children.

TE is a growing and significant problem in children with cancer. TE can be treated with anticoagulant therapy. Hence it is important to have reliable methods for early diagnosis of TE. In addition, TE is preventable with anticoagulant prophylaxis [13,14]. However, children with cancer are also at risk of bleeding from thrombocytopenia and other effects of treatment. Children with cancer receiving anticoagulation therapy or prophylaxis are shown to have 5-15% bleeding risk [9,10,56]. Ideally, thromboprophylaxis is offered only to those patients with high risk of thrombosis and low or acceptable risk of bleeding. Thus, for judicious use of thromboprophylaxis, identifying a population at high risk for TE is of paramount importance. The proposed study aims to identify predictors of TE in children with cancer.

If we confirm our primary hypothesis then we can use CVL-dysfunction as a predictor of TE and will be able to identify children at high risk for TE. Based on the results of this study we will design future studies for earlier diagnosis of TE using definitive methods like venography and future randomized controlled trial of prophylactic anticoagulant therapy in children with cancer. This will ultimately help to reduce the incidence of TE and its impact on overall outcome as well as quality of life in children undergoing treatment for cancer.

If we confirm the association of CVL-dysfunction, TE and infection, then we can identify children at high risk for infection (a major complication in children with cancer) and institute prophylactic strategies aimed at reducing dysfunction and infection (e.g. anticoagulation therapy).

In addition, this study will compare the practices of CVL insertion and care across five pediatric tertiary care centers in Ontario. Although each institution has based their practices on internal quality control studies, there are so far no data comparing or evaluating different practices across the Province in large patient population. This study will allow us to evaluate the impact of institutional practices of CVL insertion and care on the development of CVL-related complications. This will help to develop evidenced based guidelines for CVL insertion and care.

### Strengths and potential limitations of the study

The major strength of the study is the use of a prospective cohort design. Previous studies were single institution studies using retrospective design [17,32,33,36]. These studies report up to 30% of missing data [17,32,33,36]. To avoid this problem we will use uniform data collection forms for all study participants and for patients with TE which is likely to capture all the relevant information. Further, the CVL access data will be collected prospectively and hence the exposure criteria will be recorded properly avoiding any recall bias.

Unlike previous studies, evaluation of CVL-dysfunction is the primary aim of our study which formed the basis of our sample size calculation. By inclusion of only symptomatic and objectively confirmed TE, we will avoid ambiguity over diagnosis, and thus, reporting of TE. Uniform guidelines for diagnosis and evaluation will likely minimize chances of misdiagnosis of TE.

In addition the following steps are taken to minimize sources of errors and likely biases improving internal validity of the study. To avoid selection bias all consecutive newly diagnosed non-CNS cancer patients meeting the inclusion criteria will be eligible and approached for participation in the proposed study. All reported events of TE will be confirmed by an independent adjudication committee. To avoid recall bias CVL access data will be collected prospectively and hence the exposure criteria will be recorded properly. Assessors (radiologists) evaluating for TE will be blinded as regards the “exposure” status to avoid measurement bias. With the use of uniform data collection forms for all study participants and patients with TE, infection and CVL dysfunction, all the relevant information is likely to be captured. Further, all data will be analyzed centrally.

Another strength of the study is that this study is conducted across five pediatric oncology institutions with inclusion of wide range of cancer diagnoses. Hence we think that the findings of the study will be generalizable to pediatric oncology population. The results of this study will also be applicable for adults with cancer as well as to the population of patients with other chronic diseases who need long term CVL.

The main limitation is that we will not be screening for asymptomatic TE. However, the significance of asymptomatic TE detected by screening method is unknown. In addition, children with cancer have an increased risk of TE throughout the cancer-therapy making it difficult to choose an ideal time for screening [17,27,31,36]. The large amount of data collection could be perceived as a limitation of this study. However, this will allow us to evaluate various variables.

In summary, use of CVL has improved the quality of life and care in children with cancer. However, CVL is associated with significant complications namely CVL-dysfunction, TE and infection. This study aims to identify the relationship of these three CVL-related complications and evaluates the role of CVL-dysfunction as a predictor of TE in children with cancer. This study will also prospectively evaluate the impact of CVL-dysfunction on the outcome from cancer. The results of this study in turn will help to identify children at high risk for TE and to develop preventive strategies for both infection and thrombosis. This, we believe, will improve overall outcome of children with cancer.

## Competing interests

The authors declare that they have no competing interests.

## Authors’ contributions

UA conceptualize the study and wrote the grant application and has overall project responsibility. AC and LB are thrombosis expert who helped with conceptualization. KN is the central study coordinator and oversees study conduct and data collection. JH, UA and KN drafted and revised the manuscript. JH, PG, MS and LB are site investigators. They contributed to the study design, grant application and study conduct. LT is responsible for the statistical and analytic aspects of the study. All authors assisted in editing draft manuscripts and read and approved the final manuscript.

## Pre-publication history

The pre-publication history for this paper can be accessed here:

http://www.biomedcentral.com/1471-2407/12/314/prepub
